# Patient and caregiver perspectives on a tool to increase recognition of undiagnosed dementia: a qualitative study

**DOI:** 10.1186/s12877-021-02523-0

**Published:** 2021-10-26

**Authors:** Lorella Palazzo, Clarissa Hsu, Deborah E. Barnes, Marlaine Figueroa Gray, Mikael Anne Greenwood-Hickman, Eric B. Larson, Sascha Dublin

**Affiliations:** 1 Kaiser Permanente Washington Health Research Institute, 1730 Minor Avenue Suite 1600, Seattle, WA 98101 USA; 2 Departments of Psychiatry and Behavioral Sciences and Epidemiology & Biostatistics, University of California San Francisco, San Francisco, CA USA; 3 San Francisco Veterans Affairs Health Care System, San Francisco, CA USA

**Keywords:** Dementia, Focus groups, Risk assessment, Early diagnosis, Electronic health records

## Abstract

**Background:**

Early detection of dementia may improve patient care and quality of life, yet up to half of people with dementia are undiagnosed. Electronic health record (EHR) data could be used to help identify individuals at risk of having undiagnosed dementia for outreach and assessment, but acceptability to people with dementia and caregivers is unknown.

**Methods:**

We conducted five focus groups at Kaiser Permanente Washington (KPWA), an integrated healthcare system in Washington State, to explore people’s feelings about timing of dementia diagnosis, use of EHR-based tools to predict risk of undiagnosed dementia, and communication about risk. We recruited people enrolled in KPWA who had dementia or mild cognitive impairment, people enrolled in KPWA who had neither diagnosis, and caregivers (i.e., loved ones of people with dementia who assist with various tasks of daily life). People who were non-white or Hispanic were oversampled. Two team members analyzed transcripts using thematic coding.

**Results:**

Forty people (63% women; 59% non-white or Hispanic) participated in the focus groups. Themes that arose included: perceived pros and cons of early dementia diagnosis; questions and concerns about a potential tool to assess risk of undiagnosed dementia; and preferences related to patient-provider conversations disclosing that a person was at high risk to have undiagnosed dementia. Participants supported early diagnosis, describing benefits such as time to adjust to the disease, plan, involve caregivers, and identify resources. They also acknowledged the possible psychosocial toll of receiving the diagnosis. Participants supported use of an EHR-based tool, but some people worried about accuracy and privacy. Participants emphasized that information about risk of undiagnosed dementia should be communicated thoughtfully by a trusted provider and that the conversation should include advice about prognosis, treatment options and other resources when a new dementia diagnosis was made.

**Conclusion:**

People with dementia or mild cognitive impairment, people with neither diagnosis, and caregivers of people with dementia supported using EHR-based tools to help identify individuals at risk of having undiagnosed dementia. Such tools must be implemented carefully to address concerns and ensure that people living with dementia and their caregivers are adequately supported.

**Supplementary Information:**

The online version contains supplementary material available at 10.1186/s12877-021-02523-0.

## Background

In the United States, about 5.8 million people currently have dementia [1], yet a large proportion of people with dementia are undiagnosed [2–4]. However, routine screening of older adults for dementia remains controversial. Boustani et al. found that adults with and without dementia caregiving experience differed in the perceived benefits and receptivity to routine screening, with caregivers less likely to accept dementia screening [5]. In other studies, a majority of patient participants were willing to undergo dementia screening, especially if they perceived benefits to screening or were in certain age groups [2]. Physicians are divided, with some worrying about the harms of dementia screening [6], while others argue that screening may improve the lives of people with dementia overall [7, 8].

The U.S. Preventive Services Task Force (USPSTF) does not recommend for or against routine screening for dementia in older adults due to lack of evidence on benefits and harms [3]. Outside of the U.S., the UK National Institute for Health and Care Excellence (NICE) has taken a similar position [4]. However, several organizations, such as the Alzheimer’s Association and the Gerontological Society of America, advocate for early diagnosis to maximize time for planning and support for people with dementia and caregivers [5, 6].

A body of international literature documents research and practice efforts aimed at supporting early dementia diagnosis [7–9] in developed and especially in low- and middle-income countries, where the majority of people with dementia live. The Global Dementia Prevention Program (GloDePP) prioritizes developing and piloting contextually appropriate digital and artificial intelligence tools for early detection of dementia and intervention. Across countries, assessing neurophysiological signs of cognitive decline [10] and using mobile technologies (e.g. cell phone apps) for cognitive [11] evaluation are viewed as avenues to improving recognition of pre-symptomatic Alzheimer’s disease and facilitating early diagnosis [8].

As mostly responsible for comprehensive patient care and for providing first access to the health care system, primary care providers play a critical role in assessing early signs of dementia [12]. However, there are many barriers to dementia diagnosis in primary care. Dementia onset can be insidious [13], and people with dementia and caregivers (typically loved ones who assist a person with dementia) [14] often fail to recognize and seek care for mild cognitive changes [15, 16]. Clinicians may have difficulty recognizing symptoms during brief encounters with patients, particularly early in the disease process, or may focus on physical symptoms more than cognitive problems and concerns [17–19]. System-level barriers also exist such as competing priorities during clinical encounters [18].

To overcome existing barriers, several studies have called for standardized tools and information technology resources to support earlier recognition of dementia in primary care [2, 3, 18]. Electronic health record (EHR) data could be used to help identify individuals at risk of having undiagnosed dementia who could be targeted for outreach and assessment, but acceptability of this approach to primary care patients and caregivers is unknown.

We conducted a research study to address the following research questions: To what extent is use of EHR data to evaluate risk of undiagnosed dementia acceptable to patients and caregivers, and what can we learn from people with dementia and caregivers to guide design of a process for implementing an EHR-based risk score in clinical care?

## Methods

### Design and participants

#### Study design

Guided by an epistemology that centers people’s perspectives and the meanings they give to their experiences, we held five focus groups at Kaiser Permanente Washington (KPWA), an integrated healthcare system in the Pacific Northwest. Participants included people enrolled in KPWA, including people with diagnoses of dementia or mild cognitive impairment and people without such diagnoses, and caregivers – that is, loved ones who assist people with dementia with certain daily life tasks, such as managing finances, medications, or other tasks. All participants were sampled from people living in the geographic region surrounding Seattle, Washington. See the “Sampling and Recruitment” section below and Table 1 for a more detailed description of included participants.

**Table 1:** Inclusion and exclusion criteria by participant group

Patient Groups	
All patients were required to meet the following criteria: Enrolled in KPWA Aged 70–85 At least one healthcare visit at KPWA in the last 12 months Not living in a nursing home or special care facility Comfortable speaking English without a translator	
The following additional criteria were applied to specific patient groups:	
Dementia diagnosis	EHR-documented diagnosis of Alzheimer’s or any other dementia in the past 24 months No evidence in EHR of a low score on a cognitive screening test^a^ On phone interview, aware of being diagnosed with Alzheimer’s disease or dementia SPMSQ (21) score with ≤4 errors at time of phone screening
MCI diagnosis	EHR-documented diagnosis of MCI in the past 24 months No evidence in EHR of a low score on a cognitive screening test^a^ On phone interview, aware of being diagnosed with MCI SPMSQ (21) score with ≤4 errors at time of phone screening^b^
No dementia/MCI diagnosis^c^	No evidence in the EHR of a diagnosis of MCI or dementia No prescription fills for a dementia medication
Caregiver Group	
All caregivers were required to meet the following criteria: Identified by a patient in the dementia group as a caregiver OR individual from the sample without evidence of cognitive impairment who reported being a current caregiver of someone with dementia. Aged 18–85 Not living in a nursing home or special care facility Comfortable speaking English without a translator On phone interview, no self-reported problems with memory or thinking	

Abbreviations: *MCI* mild cognitive impairment, *SPMSQ* Short Portable Mental Status Questionnaire, *EHR* electronic health record, *KPWA* Kaiser Permanente Washington, *MMSE* Mini-Mental State Examination

^a^ Most patients did not have any cognitive test results recorded. If they did have results from a MMSE or Montreal Cognitive Assessment, the score had to be above 20

^b^ SPMSQ score with > 4 errors indicates more severe cognitive impairment. These patients were excluded to help ensure that focus group participants would be able to meaningfully participate

^c^ Due to ethics concerns, we were not able to screen this group for cognitive impairment as part of recruitment

All participants provided written informed consent. Study protocols were approved by the KPWA Institutional Review Board. All procedures were carried out in accordance with approved protocols and all relevant local, state, and national research guidelines and regulations.

Phenomenological theory [20] informed data collection and analysis, meaning that we sought to understand preferences for diagnosis timing and acceptability of a risk detection tool from the perspective of participants’ experiences and constructed meanings.

#### The eRADAR tool for assessing risk of having undiagnosed dementia

Our team has developed a tool called the electronic health record Risk of Alzheimer’s and Dementia Assessment Rule (eRADAR) that uses information in the EHR to identify people who may have undiagnosed dementia. Specifically, eRADAR uses key predictors such as dementia-related symptoms, healthcare utilization patterns, and dementia risk factors to calculate a risk score of predicted likelihood that the individual currently has undiagnosed dementia. The eRADAR tool was developed and validated using data from a long-running prospective cohort study set within KPWA that regularly assesses all participants for cognitive impairment and uses “gold standard” research protocols to identify incident dementia. Additional details of the eRADAR tool and its development and validation have been published previously [17].

#### Sampling and recruitment

All focus groups were conducted in person in the greater Seattle area, Washington, in June and July 2018. The five focus groups included participants sampled from four populations: 1) people enrolled in KPWA diagnosed with dementia (1 group); 2) people enrolled in KPWA diagnosed with mild cognitive impairment (MCI) (1 group); 3) people enrolled in KPWA who had no diagnosis of dementia or MCI (2 groups); and 4) caregivers of people with dementia (1 group).

We used purposive sampling [21] to recruit participants and oversampled non-white and Hispanic patients. See Table 1 for inclusion and exclusion criteria.

Potential patient participants were identified from electronic lists of people enrolled in KPWA. A total of 787 potential patient participants were sent a recruitment letter and given the option to opt out of further contact if preferred (*N* = 96 preemptively opted out). Research staff screened potential participants by phone for eligibility and interest until 10–12 participants were scheduled in each group. In total, 164 participants were screened by telephone, and 47 met eligibility criteria and agreed to participate in a focus group. The Short Portable Mental Status Questionnaire [22] was administered to people enrolled in KPWA with MCI or dementia diagnoses to exclude those with more severe cognitive impairment who we thought would not be able to meaningfully participate.

We identified caregivers for people living with dementia in two ways. First, we asked eligible dementia patients during their recruitment call to identify their caregiver(s). Second, when talking with potential participants who did not have dementia or MCI, we asked them if they identified as a current or recent caregiver for an individual with dementia. Participants received a $100 incentive and were offered transportation.

#### Data collection

Three qualitative researchers (CH, LP, MG), two of whom had extensive experience conducting focus groups, co-facilitated 90-min focus groups at two clinics in the greater Seattle metropolitan area. At least two of the three facilitators were at each focus group. One researcher served as the primary facilitator, while the co-facilitator served as the scribe and supported the primary facilitator by asking clarifying and follow up questions. Facilitators’ backgrounds and roles on the research team were shared with participants.

Group discussions were audio recorded and transcribed professionally. We developed semi-structured focus-group discussion guides, written documents that included key questions and possible prompts, and customized them as needed for the different groups. For example, all the focus group guides contained questions about experiences with dementia diagnosis, but patients and caregivers were asked to recount their actual experiences, while the group with no dementia or MCI diagnosis was asked about any experiences they might have had with loved ones and/or what they would like if they were to be diagnosed. We included a role-playing exercise for the first group that was not used in subsequent groups due to time considerations. All the discussion guides addressed experiences with and preferences for timing of dementia diagnosis; feelings and perceptions about memory loss and dementia; and acceptability and practical aspects of an EHR-based tool to assess undiagnosed dementia risk. The tool was described to participants as a potential resource that would not prove someone had dementia but could help identify people who might need more evaluation for dementia. The final discussion guides are included in Additional file 1. All facilitators used these discussion guides to ensure consistency in the topics raised during the focus groups. However, since qualitative data collection is open-ended and driven by participant experiences, there were expected differences in some of the specific topics and issues that emerged in each group.

#### Data analysis

Applying thematic coding [23], two authors (CH, LP) coded focus group transcripts using an iteratively developed coding list. An initial coding list informed by focus group questions was refined once through study team discussion and then used to code a reference transcript. The two coders each coded the transcript separately and then met to reconcile discrepancies [24, 25] The same process was applied to coding the remaining transcripts. With each transcript, the coding list was refined and expanded based on what emerged from the text.

Once coded, the data were pulled by code and reviewed to confirm key themes represented by the codes and surface more nuanced subthemes and connections. Themes and subthemes that emerged from multiple readings of the coded text were iteratively refined through team discussion [26]. The main themes that emerged from this analytic process were documented in a coding memo. The analytic process also revealed that the key themes of interest for this paper tended to cluster around the following higher-level domains, or major conceptual dimensions: preferences around diagnosis timing, acceptability of a risk detection tool, and perspectives on communication about dementia risk [27, 28].

Analysis relied on Atlas.ti (version 7.5.2). Quotations have been edited for clarity.

## Results

The five focus groups included 40 people: people enrolled in KPWA with diagnoses of dementia (*n* = 4) or MCI (*n* = 9), people enrolled in KPWA with no such diagnosis (two groups: *n* = 10, *n* = 11), and caregivers (*n* = 6). Table 2 shows participant characteristics. When looking across all participants, there were slightly more women than men and a large percentage of participants were college graduates. We overrecruited for ethnic and racial diversity and as a result had a higher proportion of non-white or Hispanic participants than in the local general population.

**Table 2:** Participant characteristics

Characteristic	*N* = 40 n (%)
Female Gender	25 (63)
Age	
55–64	1 (3)
65–74	9 (23)
75–84	26 (65)
85+	4 (10)
Race/Ethnicity^a^	
Non-Hispanic White	16 (41)
Black/African American	8 (21)
Asian-American	6 (15)
Hispanic/Latino	2 (5)
Other race or multiple races	7 (19)
Highest education completed^a^	
Some high school or GED	5 (13)
Some college or other school after high school	7 (18)
4-year degree	6 (15)
More than 4-year degree	21 (54)
Focus group composition	
Patients with dementia diagnosis	4 (10)
Patients with MCI diagnosis	9 (23)
Patients without MCI or dementia diagnosis	21 (53)
Caregivers	6 (15)

Abbreviations: *MCI* mild cognitive impairment, *GED* General Educational Diploma

^a^*N* = 39, information missing for 1 participant

The following themes arose in the analysis: perceived pros and cons of early dementia diagnosis; questions and concerns about a potential tool to assess risk of undiagnosed dementia; preferred approach to patient-provider conversations disclosing that a person was at high risk to have undiagnosed dementia; and topics considered important to cover in a patient-provider risk-assessment conversation. Themes clustered into these three domains: 1) preferences about dementia diagnosis timing (theme: perceived pros and cons of early dementia diagnosis), 2) perspectives on risk assessment for undiagnosed dementia (theme: questions and concerns about a potential tool to assess risk of undiagnosed dementia), and 3) perspectives on communication about dementia risk (themes: preferred approach to patient-provider conversations disclosing that a person was at high risk to have undiagnosed dementia; and topics considered important to cover in a patient-provider risk-assessment conversation).

This manuscript reports findings under the three domains. It is important to note that domain boundaries were constructed through analysis and theme identification but are not meant to segment aspects of the participant experience. Experiences and preferences about these topics existed on a continuum, and often, individuals expressed views in support of multiple perspectives (e.g., both pros and cons of early dementia diagnosis). For ease of interpretation and to reflect broad concepts we grouped themes into distinct categories. Quotes are attributed to participants based on their assigned number in the focus groups.

### Preferences about dementia diagnosis timing

Table 3 summarizes the pros and cons of early dementia diagnosis described by participants. Patients and caregivers stated that early diagnosis could allow the person with dementia to participate in planning for future needs while their cognitive capacities were still intact, and improve their and their family’s ability to deal with new challenges. This could mean implementing lifestyle changes to enhance the patient’s health and wellbeing and ensure safety as their cognitive status declined. Participants also expressed that early diagnosis could enable families to prepare for future caregiving responsibilities.

**Table 3:** Pros and cons of early dementia diagnosis noted by participants

Pros	
Time/ability to adjust to the disease, plan and prepare	You can plan…We can know what we should be doing, for instance, physical, social, eating carefully. The earlier you know, the more you can gear your life that way. Participant 5, MCI group You can start working through and being very careful with your time, with people, working with machinery and stuff like that…You know, being aware, slowing down because your brain is slowing down anyway. Participant 7, Dementia group
Opportunity to gather information and resources	In terms of my own physical health and others, I always want to know as much as possible at the front end. I think you do go through potentially a fatalistic, “Oh, my God, I have such and such.” And then it helps you also figure out how to get access to things to help you. Participant 2, Caregiver group You can have timely and/or early referrals to things, like physical therapy rehab for safety and walking, and that type of a thing. Participant 6, MCI group
Early awareness for caregivers	A spouse would love to be able to be a better support system, but that does involve early knowledge. When you have the best time to learn, to figure out what it is the best things that you can do, and to get some real assistance in carrying them out. Participant 6, MCI group The early diagnosis [for my loved one] was helpful for me because it put me on alert that I now have a greater responsibility to stay healthy. Participant 2, Caregiver group
Understanding and accommodating cognitive and behavioral changes	If the person is diagnosed early and the family knows of the diagnosis, then they are considered having the dementia rather than being considered a normal person with terrible behavior. Participant 1, No Diagnosis group 1 For me, an early diagnosis would have been helpful because I thought [the change] was all in my head. Participant 5, Caregiver group
Cons	
Emotional/psychological distress about prognosis	Do I really want to know that? If there’s nothing that can be helped, I don’t want to worry about…it’s really bad, but there’s nothing that anybody can do for me. So why live like that? Participant 7, No Diagnosis group 2 I guess the con would be, if it were for me, just knowing what was going to happen. Participant 10, MCI group
Negative/uncomfortable interactions	One of the disadvantages [of being diagnosed] is contacts with ill-informed people who without intention can be deeply hurtful to you. Participant 6, MCI group It’s still…a taboo type thing when you talk about short term memory loss. And people don’t like to hear it. Participant 10, MCI group

[O]ne of the pro[s] would be preparation for the family for facilities, costs, end-of-life sort of things that need to be decided by family, and also will give family an opportunity to adapt and…know…heart breaking as it is, this is what's [ahead]. – Participant 4, No Diagnosis group 1

Further, knowing the cause of behavior changes brought on by dementia could help family members understand and adjust their interactions with the patient and improve social support.

Along with many positives, participants also recognized potential drawbacks of early diagnosis. Those primarily included patient stress, anxiety, and social isolation.

A con for early diagnosis would be for the person themselves: depression, stress, anxiety.

– Participant 5, No Diagnosis group 1

For some participants, knowing that those diagnosed with dementia had a poor prognosis and few therapeutic options was a cause for distress. In addition, some people expressed the view that others’ perceptions or beliefs about dementia could damage social interactions, contributing to the hardship of an early diagnosis.

### Perspectives on risk assessment for undiagnosed dementia

Overall, participants liked the idea of an EHR-based tool for risk assessment of undiagnosed dementia, saying they would choose it for themselves or loved ones. Despite initial anxiety, they would want to know their risk of having undiagnosed dementia if this could help support earlier recognition of dementia or cognitive decline.

I think it would be a wonderful thing. If there's some problems, I want to know about it so I can -- I don't know what I can do, but it would just help me realize how things are going in my head. - Participant 7, Dementia group

Another perceived benefit was possible early intervention to slow disease progression or improve prognosis if dementia is eventually diagnosed.

I want to know what tests are available to determine where we are with this. I want to know what treatments are available so that we perhaps can slow it down. And then we do family planning…. including expenses.

–Participant 11, No Diagnosis group 1

Risk assessment for undiagnosed dementia made sense to participants who wanted to be engaged in their care and was seen as similar to routine medical testing.

I get regular blood work. I can even get on my phone and see my baseline on my blood. Now, if you come up with a tool like that for the probability or possibility of dementia or Alzheimer's, great. –Participant 6, No Diagnosis group 2

Some participants expressed mixed feelings or worried about psychological harm, and several participants who favored risk assessment acknowledged that others may hold different views, especially when using the tool and obtaining an elevated score might indicate the presence of undiagnosed dementia.

Some people don't want to believe it, you know, "No. No. No this is not happening to me." And there's people that want to know, like me…. But most people are somewhere in the middle. –Participant 7, Dementia group

Most questions and concerns that were raised focused on practical aspects of a risk-assessment tool, including how it was developed, whether there would be continued evaluation of its effectiveness, how providers would know how to use it, and who would have access to the risk scores. Worries about “false positive” results were also expressed. Risk assessment and diagnosis are intertwined processes, and it would be easy to consider a positive risk assessment to be a “false positive” that indicates the presence of dementia, when in actuality it constitutes a “flag” or alert that could lead to additional workup to determine whether dementia is truly present. These anxieties highlight how patients may conflate obtaining a risk assessment score with receiving a diagnosis (Table 4), while also revealing how distressing information that suggests the possibility of undiagnosed dementia can be.

**Table 4:** Questions and concerns about a potential dementia risk-assessment tool

Concern	
Risk calculation	I have an analytical mind, so the first thing I will want to say is, “Well, how did he determine this risk?” You know, what tests did he use so I know from what certainty there is. Participant 5, No Diagnosis group 1
Proper evaluation and use of a risk-assessment tool	[The tool] sounds intriguing… I assume there would be some ongoing evaluation of its efficacy. We’re not blinded by, oh, this is a new computer thing. But I appreciate it’s the result of human thought, labor and it deserves to be fairly evaluated as to whether it’s efficacious or not. Participant 3, MCI group [Primary care doctors] have got to be educated strongly in the tool and how it works and how to use it. Participant 8, No Diagnosis group 2
Privacy of risk data and potential labeling	If they put a risk label on you, how is that going to affect your care, and how many other unscrupulous people would get ahold of this and try to do something with this data? Because, man, if they think you’re demented or even at risk, they’re going to go after you and target you as a victim somewhere. Participant 5, No Diagnosis group 1 I would want [risk data] in my records. But other people, you know, might have that option of whether or not they wanted it to be. Because there’s some families with people with dementia, they’re really secretive. They keep it closed. Participant 11, No Diagnosis group 1
“False-positive” results	I think [the tool] would [be] good as long as they worked it out so there wouldn’t be false positives…Because that could just tear your life up too if you’re false diagnosis or indication that you may and you don’t. Participant 4, No Diagnosis group 1

### Perspectives on risk communication

We asked participants how they would want to hear that they or their loved ones had elevated risk for undiagnosed dementia. One group of people who had no diagnosis of dementia or MCI did a role-playing exercise of patient-doctor conversations. Table 5 provides participants’ preferences about approach, language, and topics for providers talking with patients about risk of undiagnosed dementia. Participants recommended that conversations occur in the context of established care relationships, ideally within primary care, and were concerned that some providers may not know how to talk about memory issues effectively without additional training. They also agreed that a skillful approach and the ready availability of additional resources could help reduce emotional and psychological distress possibly aroused by such conversations. Participants expressed the importance of clear messages around what the test can and cannot determine – if the provider is communicating the presence of elevated risk (and a recommendation for more evaluation) rather than an actual dementia diagnosis, then they must ensure this difference is understood by the patient.

**Table 5:** Preferred approach and content of patient-provider conversations about dementia risk assessment

Conversation approach	
Direct and thoughtful communication	You might have to stretch it out and take a long time…You just can’t say “You are having this problem, end of story.” You know, “We don’t know what to do to help you out, but this is it.” You have a responsibility to get the information out to them. Be kind and loving, have a nice smile on your face…I think most people will accept what you have to say. Unless you get somebody just [saying] “No, no, no, no, no,” and you have a different approach. Participant 7, Dementia group I think that [the first wording used] shouldn’t be possible dementia. But maybe [terms] like cognitive functioning evaluation or something like that, cognitive evaluation. Something so that when it’s first presented to you, it isn’t already pushing you in the “you can’t even hear what’s being said because you’re already thinking of dementia.” Participant 6, MCI group.
Provider skills and training	[Doctors] need to be able to speak about dementia so that it doesn’t set off all the fire alarms. And so, for doctors to talk about dementia, because most of us are in that age group where we’re all thinking about it, you know, they need training…They need words. Participant 5, No Diagnosis group 1
Communication in the context of	I think [risk assessment] should be at the primary care provider level. Because they’re your baseline physician. And they’re supposed to be responsible for your overall health. Participant 8, No Diagnosis group 2
an established care relationship	Your primary care physician [should talk to you] because that’s the one, hopefully, that you have a relationship with. You want [someone] who knows who you are. I don’t want psychology, somebody who only speaks medical-ese. I don’t want that. I want someone who knows who I am. Participant 5, No Diagnosis group 2
Clear distinction between risk assessment and diagnosis	[In the role-playing exercise] I was the doctor, and I kept trying to say that you may be at risk, because this was not a definitive test. And that we would have to do more follow-up and testing. Participant 5, No Diagnosis group 1
Involving family members	I did ask [my partner acting as patient in role-playing] how close her family was. Because…that would impact how personally I would proceed if I were the doctor…I might say, “Well, would you mind if I gave them a call” or…something like that. Participant 10, No Diagnosis group 1 [What was comforting was] the doctor [other participant acting as doctor in role-playing] explaining to me that I might be developing dementia, and we’re going to make an appointment with my son to come in and we will talk about this. Participant 9, No Diagnosis group 1
Topics important to cover in a clinician-patient risk-assessment conversation	
Information on dementia, treatment, and prognosis	It would be nice to have a human being in front of me talking about how this impacts me, and what can be done, and how things will end up and so forth. Participant 7, Dementia group [I will want to know] was there any treatment for it. And given this risk, how fast is it going to proceed? Participant 5, No Diagnosis group 1
Concrete support and resources	[My partner acting as a patient in role playing] was real clear that she wanted more specific information…She wanted literature, support groups for herself and possibly for family at a time when they could get there, like weekends or after work, that kind of stuff. Participant 10, No Diagnosis group 1

Considering family involvement, participants generally wanted loved ones to be involved in the conversation early to discuss results of such a risk assessment.

## Discussion

This qualitative study examined patients’ and caregivers’ perspectives on timing of dementia diagnosis and an EHR-based tool to assess risk of undiagnosed dementia. While previous work has explored patient and caregiver experiences of receiving and adjusting to dementia diagnosis [24, 25, 29], our study is the first to focus on a risk-assessment tool to support early recognition of dementia in the clinical setting.

We found that patients and caregivers favored early diagnosis for practical and social reasons but also raised concerns about the stress such diagnosis might cause. Robinson et al. [30] found that many people in their sample were initially in favor of early diagnosis of Alzheimer’s disease (the most common cause of dementia [31]) but reconsidered as they reflected that family and friends may become overprotective of them and limit their personal autonomy. These concerns find parallels in those expressed by our participants who worried about how others would react to their dementia diagnosis and how social interactions might be affected as a consequence. Similar to what emerged in our study, van den Dungen et al.’s systematic review found that ability to plan for the future was considered to be a key benefit and psychological distress a significant drawback of disclosing a dementia diagnosis [29].

Participants endorsed use of EHR information to assess risk of undiagnosed dementia and mentioned the benefits of this approach. Many people cited advantages including that it could lead to earlier knowledge of one’s disease status, allowing for engagement of family members and planning. They expressed a belief that early diagnosis would also support their receiving treatments that might improve prognosis.

However, focus group participants also worried about accuracy of the risk-assessment tool, disclosure of risk scores, and psychosocial stress. Fear of negative labeling, particularly in the case of “false positive” results indicating an elevated risk of undiagnosed disease, is a reminder that dementia carries significant social stigma [30]. The emphasis on concerns about “false positives” is significant beyond this study and may be relevant to other new developments related to dementia diagnosis, such as the growing interest in biomarker testing, which holds promise to help diagnose dementia but also can generate “false positives”.

Our findings indicate that patients need and want to learn how risk score and diagnosis differ. It is possible that having a better understanding of each concept could help alleviate some of the concerns expressed about a potential risk-assessment tool. Further, evaluation for undiagnosed dementia during routine care should be paired with safeguards to allay patients’ and caregivers’ concerns about privacy and unintended disclosure.

Participants voiced clear preferences for how risk should be communicated, including that communication occur in the context of an established relationship with a health care provider. These preferences may reflect the sensitivity of discussing risk assessment for undiagnosed dementia and subsequent results with patients and caregivers who may fear the disease and be apprehensive about available treatments. Participants expressed the importance of clear, direct messages delivered thoughtfully by providers. They wanted providers to convey information about prognosis, therapeutic options and other resources when a new dementia diagnosis was made. A communication toolkit [6] for primary care conversations about memory loss can provide guidance. However, specialized training may be needed to support providers in learning how to facilitate patient-centered conversations that involve family members, explain a complex tool and process for assessing risk of undiagnosed dementia, and offer concrete information and resources.

In general, implementing new programs is challenging in busy practices. Structural interventions, such as longer or consecutive visits, that support ongoing care with a trusted provider could ease difficult conversations while addressing symptoms and concerns.

Development and potential use of a risk-assessment tool is one of several approaches being considered to promote early diagnosis of dementia in primary care. Other examples include use of cognitive screening tools together with blood-based biomarkers to identify early-stage Alzheimer’s, regular individualized assessment of cognitive function based on patient and family history, and ongoing close collaboration between primary care providers and Alzheimer’s disease specialists [12]. Beyond the U.S., Chan et al.’s overview in the *Journal of Global Health* reports on innovative programs to enhance all stages of dementia care, including early diagnosis, by reducing service fragmentation as well as raising knowledge and awareness of dementia among clinicians and community workers [8, 32].

The present study had limitations. Focus group participants were a small, self-selected sample that may not reflect all perspectives [33]. They were highly educated and drawn from a single geographic region. Patients and some caregivers were members of an integrated health system with a focus on primary care; thus their experiences may differ from those of individuals receiving care in other contexts. In the focus groups it was sometimes difficult to engage participants in discussions of abstract notions of risk and a potential risk detection tool, though participants were very engaged and willing to share their perspectives on other topics. Because the tool was under development at the time the focus groups were held, we could only explore general acceptability of using EHR- based data to assess risk of undiagnosed dementia. With further development of the tool, we plan to investigate patient and caregiver perspectives on different cutoff points for defining “high risk” based on the model [34]. It is difficult to understand risk assessment outside of the context of a diagnosis, and we asked about both in our focus groups. Doing so might inadvertently have contributed to some participants conflating obtaining a risk score with receiving a dementia diagnosis. Lastly, these focus groups did not directly address barriers to obtaining a dementia diagnosis, such as the need for multiple visits and potentially for laboratory testing or imaging that may be expensive.

This study also had strengths. Within our healthcare system and geographic region, we solicited diverse perspectives, including input from people with and without memory loss and caregivers for people with dementia. We also included robust representation from non-white participants across a major metropolitan area, providing an opportunity for cultural differences in attitudes regarding dementia to surface.

## Conclusions

We found that patients and caregivers attribute many benefits to earlier dementia diagnosis, as well as recognizing possible risks. We also found that implementing EHR-based risk detection tools for undiagnosed dementia may be acceptable to many people. However, people expressed concerns which showed that implementing such tools will require a thoughtful approach and responsive health systems, including careful attention to how dementia risk and dementia diagnoses are communicated to patients and family members.

## Supplementary Information

**Additional file 1.**

## Data Availability

The datasets generated and analyzed during the current study are not publicly available because the participant consent form stated that only the research team would access the data, but analytic code lists are available from the corresponding author on reasonable request.

## Abbreviations

EHR: Electronic Health Record
eRADAR: Electronic Risk of Alzheimer’s and Dementia Assessment Rule
KPWA: Kaiser Permanente Washington
MCI: Mild Cognitive Impairment
