# Supplementary material for: Patient and caregiver perspectives on a tool to increase recognition of undiagnosed dementia: a qualitative study
Source: BMC Geriatr. 2021 Oct 26;21:604. doi: 10.1186/s12877-021-02523-0 (PMC8549374; doi:10.1186/s12877-021-02523-0)
Supplement: Supplementary file 1 — Additional file 1. [file 12877_2021_2523_MOESM1_ESM.pdf]

Appendix: Focus Group Discussion Guides

<u>Guide 1: KPWA patients diagnosed with dementia (used for 1 group)</u>

1. When I say the word “dementia,” what does that mean to you? What about the term “mild cognitive impairment”?
   a. What images or other words come up for you when I say “dementia”/ “mild cognitive impairment”?
   b. What feelings do these words bring up for you?

   You’ve mentioned some important aspects of dementia. Here are some additional things we want to make sure we are all on the same page about when we talk about dementia:
   - Dementia refers to problems with memory and thinking that interfere with daily functioning.
   - Symptoms can range from very mild to severe and may get worse over time.
   - Alzheimer’s disease is the most common cause of dementia
   - People with mild cognitive impairment have problems with memory and thinking, but these problems don’t affect their daily functioning. These people are more likely to develop dementia, but not all of them do

   Today we will be using the term “dementia” to refer to this diagnosis. Is this ok with everyone?

2. Tell us the story about getting your diagnosis of *dementia*?
   a. Tell us what led up to you getting that diagnosis?
   b. When you found out, what were your thoughts?
   c. What made getting your dementia diagnosis easier? What made it harder?
   d. What types of tests or medical appointments did you have to get your diagnosis?
   e. What information or support was most helpful to you?

3. Do you feel that you got your diagnosis at the right time?
   a. Did you get it too soon?
   b. Did you get it too late?

4. Based on your experience, would it be better to get diagnosed early?
   a. What is helpful about being diagnosed early? *<SCRIBE>*
   b. What is hard about being diagnosed early? *<SCRIBE>*
   c. Can you think of anything not already mentioned that would be helpful about getting a late diagnosis?

5. If your primary care doctor had a way to look in your medical record for clues, for example missed clinic visits, unfilled medications, diagnosis of other conditions that make you higher risk, and find people that might have *dementia* that has not been diagnosed yet. What questions do you have about a tool like this?

While this tool would not prove that someone had dementia, it would help identify people who might benefit from more testing to see whether they might have *dementia.* After the testing we would know if you had dementia or not.

   a. How might this be helpful to patients?
   b. How might this be harmful to patients?
   c. If we had this tool, would you want your doctor to use it for you?

6. Let's say this tool found someone who might have *dementia* that wasn't diagnosed yet. How should Kaiser tell that person they might have dementia?
   a. Who should follow up? (PCP, nurse, social worker, someone else?)
   b. when they come in for their next regular visit?
   c. a letter from your doctor?
   d. a phone conversation with someone at your doctor's office?
   e. Have their doctor set up a new appointment?

7. Once someone has had this conversation about being at risk for dementia, what do you think should happen next?
   a. Describe what you think the process should be for the follow-up and evaluation?
   b. How likely do you think you would be to go in for evaluation?
   c. What would the barriers be to getting further evaluation?

8. How should the patient's family be engaged in the process?
   a. What if your care team wanted a family member to come in with you. Would you be comfortable with that?

9. Let's say this person got through this process and found out they have dementia, what resources and types of care would they need?
   a. What types of help do you think you would need?
   b. What additional information would you want?
   c. Are there any other types of support groups or support strategies that you think might be helpful?

10. What other comments do you have about this topic?

Guide 2: KPWA patients diagnosed with mild cognitive impairment (MCI) (used for 1 group)

1. When I say the words "mild cognitive impairment," what does that mean to you?
    a. What images or other words come up for you when I say "mild cognitive impairment"?
    b. What feelings do these words bring up for you?

2. What about the term "dementia"?
    a. What images or other words come up for you when I say "dementia"?
    b. What feelings do these words bring up for you?

You've mentioned some important aspects of dementia and mild cognitive impairment. Here are some additional things we want to make sure we are all on the same page about when we talk about dementia:

- People with mild cognitive impairment have problems with memory and thinking, but these problems don't affect their daily functioning. Some of these people go on to get dementia, but not all of them do
- Dementia refers to problems with memory and thinking that interfere with daily functioning.
- Symptoms can range from very mild to severe, and may get worse over time.
- Alzheimer's disease is the most common cause of dementia

Today we are going to use the term ***"mild cognitive impairment"*** or the abbreviation MCI to talk about the kind of memory problems you are experiencing. Is this ok with everyone? Does anyone have any questions or concerns with using that phrase or abbreviation for our discussion today?

3. Tell us the story about talking to your doctor about your mild cognitive impairment.
    a. What led to that discussion with your doctor?
        - What types of evaluation processes (testing, specialist appointments etc.) did you go through to arrive at your diagnosis?
        - When you first found out, what were your first thoughts or concerns?
    b. What did your doctor tell you about MCI?
    c. What did your doctor do or say that was helpful or comforting to you?
    Did your doctor do or say anything that made it harder?
    d. What did your doctor say to you, if anything, about what the future might hold for you with your mild cognitive impairment?
    e. What resources were most helpful to you?
    Where did you find information and support?

4. Repeat: People with mild cognitive impairment have problems with memory and thinking, but these problems don't affect their daily functioning. Some of these people go on to get dementia, but not all of them do

Some people with dementia have memory problems or other symptoms for a long time before

getting a diagnosis from their doctor. Others may get diagnosed more quickly. Let's talk about the pro and cons of early diagnosis versus later diagnosis. We'll brainstorm these as a group.

a. What would be the pros of a late diagnosis? <*SCRIBE*>
b. What would be the cons of a late diagnosis? <*SCRIBE*>
c. What would be the pros of an early diagnosis? <*SCRIBE*>
d. What would be the cons of an early diagnosis? <*SCRIBE*>
e. For you, do you think you would have a preference of an earlier or later diagnosis?

5. Imagine your doctor had a way he or she could look for clues in your medical record to find people that are likely to have dementia that has not been diagnosed yet. For example, an algorithm could group missed visits, forgotten medication refills, having other health conditions that might increase dementia risk (e.g. stroke, etc.). While this tool would not prove that someone had dementia, it would help identify people who might benefit from more evaluation for dementia. After the evaluation some would be found to have dementia, others wouldn't.
    a. What do you think about a tool like this?
    b. Is this a good idea? Why or why not?
    c. How might this be helpful to patients?
    d. How might this be harmful to patients?
    e. If we had this tool, would you even want this used for you?
    f. Would you want to be able to opt out?
    g. There are different places a tool like this could be used, such as in primary care, when being admitted to the hospital, when being seen by a specialist, etc. What health care settings would work best for a tool like this? Why those settings and not others?
    h. Are there any settings where you think a tool like this should NOT be used?

6. Let's say the tool flagged you as being at risk for having dementia. How would you want to be contacted and told about this possibility?
    a. when you come in for your next regular visit?
    b. a letter?
    c. a phone conversation with provider?
    d. Have your provider reach out to set up a new appointment?
    e. Other things we haven't mentioned?

7. Who would you want to have this conversation with?
    a. Your PCP?
    b. A nurse?
    c. A social worker?
    d. Someone else from your PCPs office?
    e. Is there anyone else you think might be the right person to contact you?

8. What would you want to know about the tool itself?
    a. Would you be interested in knowing how you were flagged?
    b. Would you want to know how likely it was you actually had dementia?

9. Once you have this conversation with your doctor about being at risk for dementia, what do you think should happen next?
    a. What do you think should be the process for the follow-up and evaluation?
    b. How likely do you think you would be to go in for evaluation?
    c. What would the barriers be to you getting further evaluation?

10. How should family or other friends or loved ones be engaged in the process?
    a. What if your care team wanted a family member to come in with you. Would you be comfortable with that?

11. Let’s say you got through this process and found out you have dementia, what resources and types of care would you want to have in place?
    a. What types of help do you think you would need?
    b. What additional information would you want?
    c. Are there any other types of groups or support strategies that you think might be helpful?

12. For those of you who have had a close friend or relative experience dementia—what resources were most helpful to you or loved one? Where did you find information and support?

13. What other comments do you have about this topic?

14. Why did you participate in this group today?

<u>Guide 3: KPWA patients with no diagnosis of dementia or MCI (used for 2 groups)</u>

1. When I say the word "dementia," what does that mean to you?

   <allow response>
   You've mentioned some important aspects of dementia. Here are some additional things we want to say to make sure we are all on the same page about when we talk about dementia:
   - Dementia refers to problems with memory and thinking that interfere with daily functioning.
   - Symptoms can range from very mild to severe, and may get worse over time.
   - Alzheimer's disease is the most common cause of dementia
   - People with mild cognitive impairment have problems with memory and thinking, but these problems don't affect their daily functioning. These people are more likely to develop dementia, <u>but not all of them do</u>

2. What experiences have you had with someone close to you developing dementia?
   a. In your observation, what was the process of diagnosis like for them?
   b. What have you learned about how to approach the diagnosis of dementia?
      - Tell us about the process of your loved one getting their diagnosis. Were there things that went well? Things that didn't go well?
   c. What have you learned about the services and resources needed once someone is diagnosed with dementia? *(affirm and bring back)*
   d. How do these experiences shape your thoughts and concerns about dementia?

3. We want to hear about how you might respond if you were told that you may have dementia. So, imagine you have just met with your doctor, who has expressed that they are concerned that you may have dementia. I'm going to give you a half a minute or so to draw or write words that come to mind for you in this scenario.
   a. What did you write or draw? <*SCRIBE*>

4. Now I want to expand on these initial reactions. What other thoughts, questions or concerns come up when you think about developing dementia?

5. For some people with dementia, they may have memory problems or other symptoms for a long time before getting a diagnosis from their doctor. For others, they may get diagnosed more quickly. Next, we are going to discuss the pro and cons of early versus later diagnosis. That is, the pros and cons of being told you have dementia when the disease is at earlier stage versus later, when it has advanced and become more severe. We'll brainstorm these as a group.
   a. What would be the pros of a late diagnosis? <*SCRIBE*>
   b. What would be the cons of a late diagnosis? <*SCRIBE*>
   c. What would be the pros of an early diagnosis? <*SCRIBE*>
   d. What would be the cons of an early diagnosis? <*SCRIBE*>
   e. Do you think you would have a preference of an earlier or later diagnosis?

6. Imagine your doctor had a way he or she could look for clues in your medical record to find people that are likely to have dementia that has not been diagnosed yet. For example, missed visits, forgotten medication refills, having other health conditions that might increase dementia risk (e.g. stroke, etc.). While this tool would not prove that someone had dementia, it would help identify people who might benefit from more evaluation for dementia. After the evaluation some would have dementia, others wouldn't.
   a. What do you think about a tool like this?
   b. How might this be helpful to patients?
   c. How might this be harmful to patients?
   d. If we had this tool, would you even want this used for you?
   e. Would you want to be able to opt out?
   f. There are different places a tool like this could be used, such as in primary care, when being admitted to the hospital, when being seen by a specialist, etc. What health care settings would work best for a tool like this?
   g. Are there any settings where you think a tool like this should NOT be used?

7. Let's say the tool flagged you as maybe having undiagnosed dementia. How would you want to be contacted and told about this possibility? For example:
   a. when you come in for your next regular visit?
   b. a letter?
   c. a phone conversation with provider?
   d. Have your provider reach out to set up a new appointment?
   e. Other things we haven't mentioned?

8. Who would you want to have this conversation with?
   a. Your PCP?
   b. A nurse?
   c. A social worker?
   d. Someone else from your PCPs office?
   e. Is there anyone else you think might be the right person to contact you?

9. What would you want to know about the tool itself?
   a. Would you be interested in knowing <u>how you were flagged</u>?
   b. Would you want to know <u>how likely</u> it was you actually had dementia?

10. Next we want your thoughts about how best to let a patient know that he/she might have undiagnosed dementia.

    Key questions we have are: What should your doctor tell you? How could this message be phrased so that it is not too upsetting? What words should be used?

    a. Exercise Round 1 (*instructions written on flipchart beforehand*): To get at this we are going to ask you to role play in groups of <u>three</u> (one person can take notes) an exchange between a doctor-patient about this topic. We have a short script with some language that other people like you have come up with that they thought would be helpful. We

would like you to try out this language in your group and note your reactions to it and any changes you would make. Decide who will be the doctor, who will be the patient, and who will take notes. As you use the script, I want you to note your feelings, what comes to mind, and how the experience could be improved:

- What are your reactions to either giving or receiving the information using the language we provided?
- What changes would you make? <10 MIN. TO ROLE PLAY>
- OK, let's talk about those role plays and what notes you have taken
    1. How did you feel either giving or getting the information about possible dementia?
    2. What words or phrases were particularly comforting or reassuring in the original? What changes did you make? *<SCRIBE>*
    3. What words or phrases were alarming in the original? What changes did you make*<SCRIBE>*
    4. What other thoughts or reactions did you have?

SCRIPT

"So wonderful to see you today. As your doctor, I want to help you manage your health in a way that lets you remain as independent and active as possible. Sometimes we use screening systems to alert us to the possibility that patients are at increased risk for health problems. One of those screening systems has alerted me that you might be at risk for memory or thinking challenges. I am not saying you have this type of condition, only that we might want to discuss this issue more and possibly do more testing to assess this situation. What are your thoughts about doing some additional testing in this area? If you wish, you might want to have a family member or friend come with you to your next appointment. Are you interested in involving family at this point?"

b. Exercise Round 2 (*instructions written on flipchart beforehand*): Now I'd like to switch the doctor-patient roles and try out the modified script that you came up with.
- What was comforting/reassuring this time around? *<SCRIBE?>*
- What still raised concerns for you? <SCRIBE?>
- What other thoughts or reactions did you have?

11. Once you have this conversation about being at risk for dementia, what do you think should happen next?
    a. Describe what you think the process should be for the follow-up and evaluation
    b. How likely do you think you would be to go in for evaluation?
    c. What would the barriers to getting further evaluation?

12. How should family or other friends or loved ones be engaged in the process?
    a. What if your care team wanted a family member to come in with you?
    b. Would you be comfortable with that?

13. Let's say you got through this process and found out you have dementia. What resources and types of care would you want to have in place?
    a. What types of help do you think you would need?
    b. What additional information would you want?

c. Are there any other support strategies (e.g. support groups) that you think might be helpful?

14. For those of you who have had a close friend or relative experience dementia:
    a. What resources were most helpful to you or loved one?
    b. Where did you find information and support?

15. What other comments do you have about this topic?

<u>Guide 4: Caregivers of KPWA patients with dementia (used for 1 group)</u>

1. When I say the word "dementia," what does that mean to you?
   <allow response>
   You've mentioned some important aspects of dementia. Here are some additional things we want to say to make sure we are all on the same page about when we talk about dementia:
   - Dementia refers to problems with memory and thinking that interfere with daily functioning.
   - Symptoms can range from very mild to severe, and may get worse over time.
   - Alzheimer's disease is the most common cause of dementia

2. In this group, we want to hear from people who have helped a loved one through the process of being diagnosed with dementia or another memory/thinking challenge. All of you are here because you were identified as a loved one and/or caregiver for someone in this situation. We would like each of you to briefly share your story of your loved one's diagnosis.
   a. Can you talk about what led up to your loved one getting that diagnosis?
   b. At what point were you, as the caregiver, brought into the conversation about your loved one's diagnosis and care?
   c. What did the process of getting diagnosed look like?
   d. What was the most helpful part of this process?
   e. What was the least helpful?
   f. What did the person's doctor do or say that made it easier to hear this diagnosis? Made it harder?
   g. How did you feel about the timing of their diagnosis? Did it feel too early? Too late?
   h. From what you observed, what was the experience of getting diagnosed like from your loved one's perspective?

3. For some people with dementia, they may have memory or thinking problems or other symptoms for a long time before getting a diagnosis from their doctor. For others, they may get diagnosed more quickly. Next, we are going to discuss the pro and cons of early versus later diagnosis. We'll brainstorm these as a group.
   a. What would be the pros of an early diagnosis? <*SCRIBE*>
   b. What would be the cons of an early diagnosis? <*SCRIBE*>
   c. What would be the pros of a late diagnosis? <*SCRIBE*>
   d. What would be the cons of a late diagnosis? <*SCRIBE*>
   e. Thinking of your loved one's experiences, do you think you would have a preference of an earlier or later diagnosis?

4. Imagine that doctors had a way he or she could look for clues in patients' medical records to find people that are likely to have dementia that had not been diagnosed yet. For example, missed visits, forgotten medication refills, having other health conditions that might increase dementia risk (e.g. stroke, etc.). While this tool would not prove that someone had dementia, it could help identify people who might need more evaluation for dementia. After the evaluation some would have dementia, others wouldn't. What questions would you have about a tool like this?
   a. Is this a good idea? Why or why not?

b. How might this have been helpful to your loved one or to patients in general?
c. How might this have been harmful to your loved one or to patients in general?
d. If we had this tool, would you have wanted this used for your loved one?
e. Would you want to be able to opt out?
f. There are different places a tool like this could be used. How would you feel about you loved one's primary care doctor using a tool like this? What if your loved one were being admitted to the hospital, should it be used there? What about when being seen by a specialist, etc.? What health care settings would work best for a tool like this? Why those settings and not others?
g. Are there any settings where you think a tool like this should NOT be used?

5. Let's say the tool flagged your loved one as being at risk for having dementia. How would you want them to be contacted and told about this possibility?
   a. When they come in for their next regular visit?
   b. A letter?
   c. A phone conversation with provider?
   d. Should the provider reach out to the person to set up a new appointment?
   e. Other things we haven't mentioned?

6. Who should be the main contact person to follow-up with a person who might have dementia?
   a. PCP?
   b. A nurse?
   c. A social worker?
   d. Someone else from PCPs office?
   e. Is there anyone else you think might be the right person to make contact?

7. How should you as loved ones/caregivers be engaged in the process?
   a. What conversations are particularly important for loved ones to participate in?
   b. Based on your experience, what might the challenges be of engaging loved ones in the diagnosis process?

8. Once you or your loved one had this conversation about being at risk for dementia, what do you think should happen next with regard to follow up and evaluation?
   a. Describe what you think the process should be for the follow-up and evaluation?
   b. What would the barriers be to getting further evaluation?

9. Let's say you got through this process and found out your loved one has dementia, what resources and types of care would you want to have in place for them as a patient and you as a caregiver?
   a. What types of help do you think you would need?
   b. What additional information would you want?
   c. Are there any other types of groups or support strategies that you think might be helpful?

10. Since all of you have had a loved one experience memory problems or dementia—what resources were most helpful to you? Where did you find information and support?

a. What have you learned about how to care for someone with dementia?
b. Tell us about the process of your loved one getting their diagnosis. Were there things that went well? Things that didn't go well?
c. What have you learned about the services and resources needed once someone is diagnosed with dementia?

11. How would you feel about a tool like this being used for you?

12. What other comments do you have about this topic?
